# Big data research is everyone's research—Making epilepsy data science accessible to the global community: Report of the ILAE big data commission

**DOI:** 10.1002/epd2.20288

**Published:** 2024-10-24

**Authors:** Colin B. Josephson, Eleonora Aronica, Sandor Beniczky, Danielle Boyce, Gianpiero Cavalleri, Spiros Denaxas, Jacqueline French, Lara Jehi, Hyunyong Koh, Patrick Kwan, Carrie McDonald, James W. Mitchell, Stefan Rampp, Lynette Sadleir, Sanjay M. Sisodiya, Irene Wang, Samuel Wiebe, Clarissa Yasuda, Brett Youngerman, the ILAE Big Data Commission

**Affiliations:** ^1^ Department of Clinical Neurosciences, Cumming School of Medicine University of Calgary Calgary Alberta Canada; ^2^ Hotchkiss Brain Institute University of Calgary Calgary Alberta Canada; ^3^ Department of Community Health Sciences, Cumming School of Medicine University of Calgary Alberta Canada; ^4^ O'Brien Institute for Public Health University of Calgary Calgary Alberta Canada; ^5^ Centre for Health Informatics University of Calgary Calgary Alberta Canada; ^6^ Institute for Health Informatics University College London London UK; ^7^ Department of (Neuro)Pathology, Amsterdam UMC University of Amsterdam, Amsterdam Neuroscience Amsterdam The Netherlands; ^8^ Stichting Epilepsie Instellingen Nederland (SEIN) Heemstede The Netherlands; ^9^ Department of Neurology, Albert Szent‐Györgyi Medical School University of Szeged Szeged Hungary; ^10^ Department of Neurophysiology Danish Epilepsy Center Dianalund Denmark; ^11^ Department of Clinical Medicine, Aarhus University and Department of Clinical Neurophysiology Aarhus University Hospital Aarhus Denmark; ^12^ Tufts University School of Medicine Boston Massachusetts USA; ^13^ Johns Hopkins University Biomedical Informatics and Data Science Section Baltimore Maryland USA; ^14^ West Chester University Department of Public Policy and Administration West Chester Pennsylvania USA; ^15^ School of Pharmacy and Biomolecular Sciences The Royal College of Surgeons in Ireland Dublin Ireland; ^16^ FutureNeuro SFI Research Centre The Royal College of Surgeons in Ireland Dublin Ireland; ^17^ British Heart Foundation Data Science Center Health Data Research UK London UK; ^18^ Department of Neurology Grossman School of Medicine, New York University New York New York USA; ^19^ Epilepsy Center Cleveland Clinic Cleveland Ohio USA; ^20^ Center for Computational Life Sciences Cleveland Ohio USA; ^21^ Harvard Brain Science Initiative Harvard University Boston Massachusetts USA; ^22^ Department of Neuroscience, School of Translational Medicine Monash University Melbourne Victoria Australia; ^23^ Department of Neurology Alfred Health Melbourne Victoria Australia; ^24^ Department of Neurology The Royal Melbourne Hospital Parkville Victoria Australia; ^25^ Department of Radiation Medicine and Applied Sciences & Psychiatry University of California San Diego California USA; ^26^ Institute of Systems, Molecular and Integrative Biology (ISMIB) University of Liverpool Liverpool UK; ^27^ Department of Neurology The Walton Cetnre NHS Foundation Trust Liverpool UK; ^28^ Department of Neurosurgery and Department of Neuroradiology, University Hospital Erlangen, Department of Neurosurgery University Hospital Halle (Saale) Halle (Saale) Germany; ^29^ Department of Paediatrics and Child Health University of Otago Wellington New Zealand; ^30^ Department of Clinical and Experimental Epilepsy, UCL Queen Square Institute of Neurology London WC1N 3BG and Chalfont Centre for Epilepsy London UK; ^31^ Epilepsy Center, Neurological Institute Cleveland Clinic Cleveland Ohio USA; ^32^ Clinical Research Unit, Cumming School of Medicine University of Calgary Calgary Alberta Canada; ^33^ Department of Neurology University of Campinas Campinas Brazil; ^34^ Department of Neurological Surgery Columbia University Vagelos College of Physicians and Surgeons New York New York USA

**Keywords:** artificial intelligence, big data, common data models, epilepsy, ethics

## Abstract

Epilepsy care generates multiple sources of high‐dimensional data, including clinical, imaging, electroencephalographic, genomic, and neuropsychological information, that are collected routinely to establish the diagnosis and guide management. Thanks to high‐performance computing, sophisticated graphics processing units, and advanced analytics, we are now on the cusp of being able to use these data to significantly improve individualized care for people with epilepsy. Despite this, many clinicians, health care providers, and people with epilepsy are apprehensive about implementing Big Data and accompanying technologies such as artificial intelligence (AI). Practical, ethical, privacy, and climate issues represent real and enduring concerns that have yet to be completely resolved. Similarly, Big Data and AI‐related biases have the potential to exacerbate local and global disparities. These are highly germane concerns to the field of epilepsy, given its high burden in developing nations and areas of socioeconomic deprivation. This educational paper from the International League Against Epilepsy's (ILAE) Big Data Commission aims to help clinicians caring for people with epilepsy become familiar with how Big Data is collected and processed, how they are applied to studies using AI, and outline the immense potential positive impact Big Data can have on diagnosis and management.

**Key points**

- Big Data and advanced analytics are increasingly becoming a foundational to routine medical care, especially epilepsy since the diagnosis and management of the disease generates multiple sources of high‐dimensional data.
- Big Data generation and analysis should be available to all centers and interested parties, and fundamental approaches to demystify and democratize access are outlined in this paper.
- Health care professionals should be familiar with common sources of harmful bias, suboptimal validation, risks of disparity, and climate impacts of Big Data and advanced analytics.
- Generation and analysis of big data must be inclusive, incorporating views from researchers, health care providers, policy makers, and the global community of people affected by epilepsy.
- By incorporating a systematic and deliberate approach, Big Data and advanced analytics have the potential to revolutionize how we manage epilepsy across the globe.

## 1 INTRODUCTION

Big Data is rapidly becoming part of our collective consciousness. Early attempts to define this conceptually involved data visualization and the barriers presented by the digitization of large volumes of data. Its definition later progressed to comprise a series of defining “Vs,” initially the “volume” of data, the “velocity” with which data are produced, and the “variety” of data available. This has further expanded to include the “veracity” and “value” of data^1^, and additional “V's,” such as “volatility,” “variability,” and “visualization,” have been proposed to further broaden the scope. Although a multitude of formal definitions exist^1^, some argue that the concept of Big Data now extends beyond these constraints and that it should additionally include data that are “useful and can be reused, accumulate value over time, and innovate multi‐dimensional, systems‐level understanding.”^2^

Due to the heterogeneity of etiologies and syndromes, epilepsy may simultaneously produce and derive disproportionate benefit from Big Data. To properly characterize seizure types, epilepsy types, epilepsy syndromes, etiologies, and comorbidities,^3^ people with epilepsy often require a myriad of diagnostic tests including, but not limited to, electroencephalography (EEG), magnetic resonance imaging (MRI), and genetic testing, sometimes more than once. As an example, the volume of digital EEG produced since its inception now easily exceeds a petabyte (2^50^ bytes) of information.^4^ In combination with the growing expanse of clinical, imaging, biophysical, and genetic data, a rich and comprehensive picture of individuals with epilepsy, including their unique disease trajectories, can be realized if we are able to amalgamate these data sources that frequently remain siloed. This is critically important since this heterogeneity also means that smaller studies may only represent a specific subset of those with the disease, rendering the results applicable to only specific syndromes or epilepsy types. Big Data can provide a catalyst to best characterize these rarer forms of epilepsy, providing sufficient information to comprehensively describe the disease state, response to treatment, and prognosis.

Big Data and AI will form an increasingly central role in the provision of medicine over the coming years and decades. Proprietary platforms, such as IBM Watson and Google's Deep Mind, are currently being used to advance diabetes and cancer management, identify novel drugs, and improve diagnostic flow.^5^, ^6^ These approaches will be used in epilepsy, and thus we are at a juncture where it is critical to ensure people with epilepsy and their care providers are familiar with Big Data and advanced analytics.

This paper constitutes the educational component of the International League Against Epilepsy's (ILAE) Big Data Commission's white paper series on the dissemination, democratization, and equitable application of Big Data in epilepsy. It focuses on the general sources of Big Data in epilepsy, with overviews of how we derive clinical, electroencephalographic, imaging, neuropsychological, and genetic information. It then reviews how these data can be linked and leveraged by advanced techniques such as machine learning and artificial intelligence (AI). After describing the fundamental processes, the paper then discusses contemporary issues in Big Data and AI, including ethics, societal engagement, and environmental impact. The paper then concludes by addressing the current barriers to Big Data uptake and the opportunities that lie ahead.

## 2 BIG DATA IN EPILEPSY

### 2.1 The landscape of epilepsy‐related data

#### 2.1.1 Clinical

**FIGURE 1:**
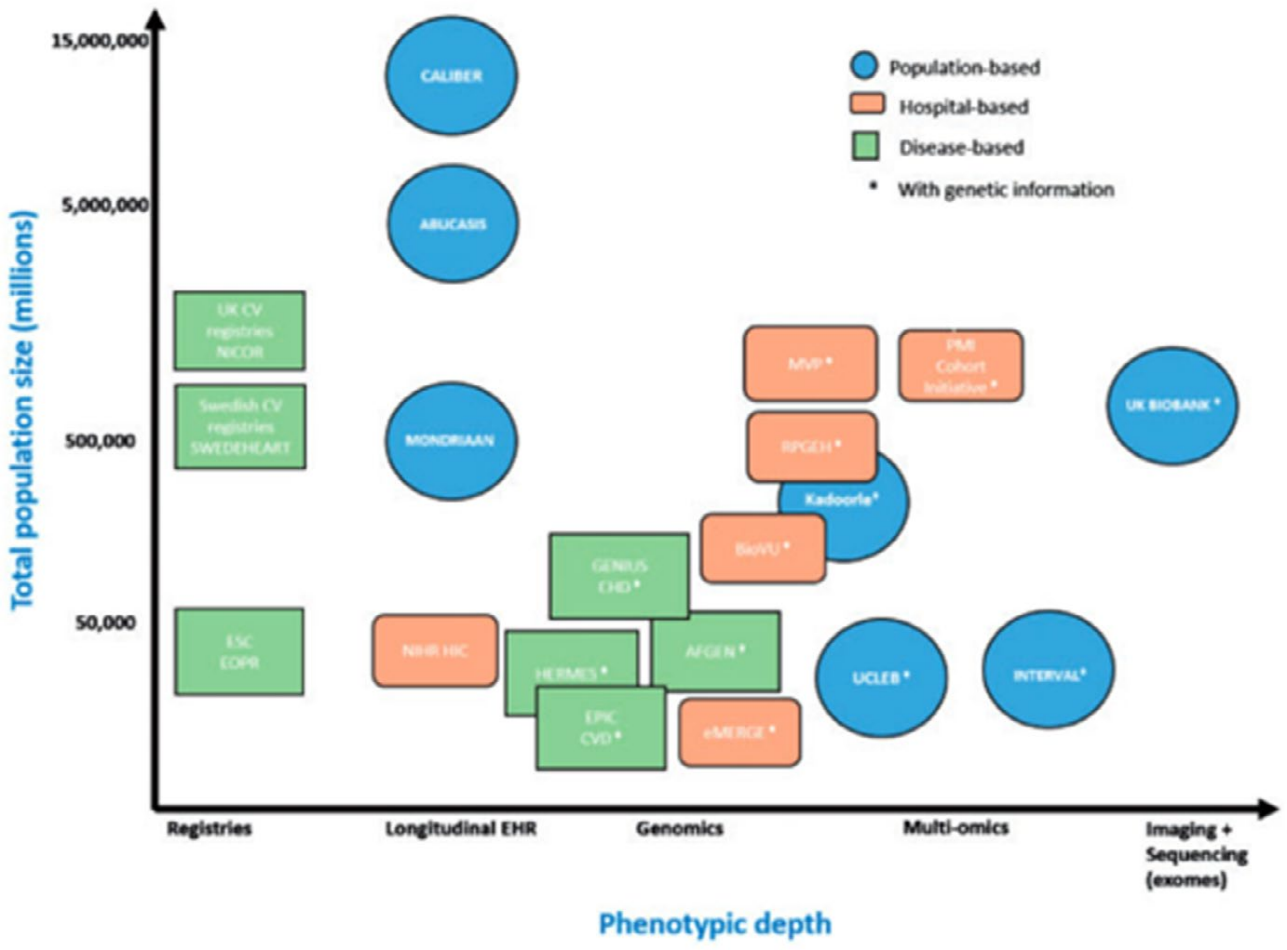
Manifold sources of clinical data now exist, with inherent trade‐offs between population size, phenotypic depth, ease of access, and complexity of database infrastructure and relationships. Reproduced from.^117^ AF, atrial fibrillation; AFGen, AF Consortium; CHD, coronary heart disease; eMERGE, Electronic Medical Records and Genomics; EPIC, European Prospective Investigation into Cancer and Nutrition; ERFC, Emerging Risk Factors Collaboration; ESC, European Society of Cardiology; HF, heart failure; MVP, Million Veterans Programme; NICOR, National Institute for Cardiovascular Outcomes Research; NIHR, National Institute for Health Research; PMI, precision medicine initiative; RPGEH, Research Programme on Genes, Environment, and Health; UCLEB, University College, London School of Hygiene and Tropical Medicine, Edinburgh, Bristol.

Coinciding with the advent of the routine collection of administrative health records (AHRs), the expansion of electronic health records (EHRs)/electronic medical records (EMRs) systems, and greater uptake of disease registries, clinical data is accruing at an unprecedented rate (Figure 1). The value of these data is self‐evident given the longitudinal demographic, socioeconomic, diagnostic, therapeutic, and health systems information they contain. However, these data are not without limitations. The size and long‐term traction of registries are frequently restricted by human and financial resources and the arduous nature of data entry. Data derived from AHR and EHR systems offer vastly larger populations, but both are highly complex, comprising structured, semi‐structured, and unstructured data. These repositories may also have varying levels of completeness, accuracy, complexity, and bias.

To address the problem of data accuracy, the ILAE is undertaking three major tasks. Firstly, through position statements, they aim to promote consistent use of epilepsy concepts and terminology by clinicians. Secondly, they provide professional development and educational courses to help disseminate this information so that medical and allied health providers from all levels and specialties can accurately diagnose epilepsy and speak the same ‘epilepsy language’. Thirdly, the ILAE is actively working with organizations that develop classification and ontology used globally for data coding, such as the International Classification of Disease (ICD) and Systematized Nomenclature of Medicine Clinical Terms (SNOMED‐CT), to ensure these concepts are consistent with the ILAE conceptual framework. These terms can be combined with pharmaceutical (e.g., RxNorm) and procedural (e.g., Office of Population Censuses and Surveys Classification of Interventions and Procedures, Current Procedural Terminology) codes in an iterative fashion to define EHR/AHR “phenotypes” of epilepsy that can be used for surveillance and research purposes if acceptable levels of accuracy are achieved.^7^, ^8^, ^9^

**FIGURE 2:**
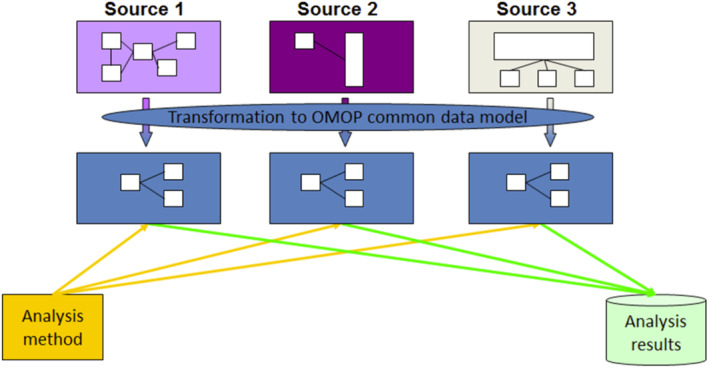
The Observational Medical Outcomes Partnership (OMOP) Common Data Model (CDM) can accommodate both administrative claims and electronic health records, allowing users to generate evidence from a wide variety of sources. It can also support collaborative research across data sources both within and outside the United States, in addition to being manageable for data owners and useful for data users. Once a database has been converted into the OMOP CDM, evidence can be generated using standardized analytics tools.

Given the evident complexities of these data, minimum standards for EHR research (CODE‐EHR) now exist that promote transparent reporting for data coding, linkage, case definitions, analytic approaches, and governance.^10^ These are critical since Big Data research ultimately relies on the quality of data entered into EHR/AHR and registry systems. Efforts at creating common data models (CDMs) have also been developed and are continually evolving to further facilitate these surveillance and research efforts. CDMs represent ways of organizing data into standard structures that promote data homogenization across institutions and regions.^11^ An exemplar of this approach is the Observational Health Data Sciences and Informatics' (OHDSI) Observational Medical Outcomes Partnership (OMOP) CDM.^12^ Originally published in 2007, OMOP uses a platform‐agnostic data modeling approach that employs a fixed and rigid structure of finite tables containing standardized concepts and vocabularies organized hierarchically (Figure 2).^13^, ^14^ Efforts, including the Fast Healthcare Interoperability Resources (FHIR), have also been made to produce rules and protocols that enable quick and secure data exchange and homogenization across independent health information systems.^15^ These approaches promote pooled (central data storage at a single lead site) and federated (local data storage at each participating site) data analyses,^11^ thus propelling Big Data studies from the world of the theoretical to the practical.

#### 2.1.2 EEG

EEG measures differences in electric potentials generated mainly by the brain's neuronal activity. A magnetoencephalography is a related approach that measures the magnetic correlates of brain activity. These modalities provide functional insights into brain activity with very high temporal resolution in the millisecond range. An EEG is important in diagnosing epilepsy, especially when the clinical information about the seizures is less clear and in people with only one unprovoked seizure. An EEG also helps to classify the type of epilepsy and hence, choose optimal therapy. Apart from MRI, EEG is the most important modality for presurgical evaluation of people with drug‐resistant focal epilepsy,^16^, ^17^ and invasive EEG is often used to identify and delineate an area for surgical resection in complex cases.

**FIGURE 3:**
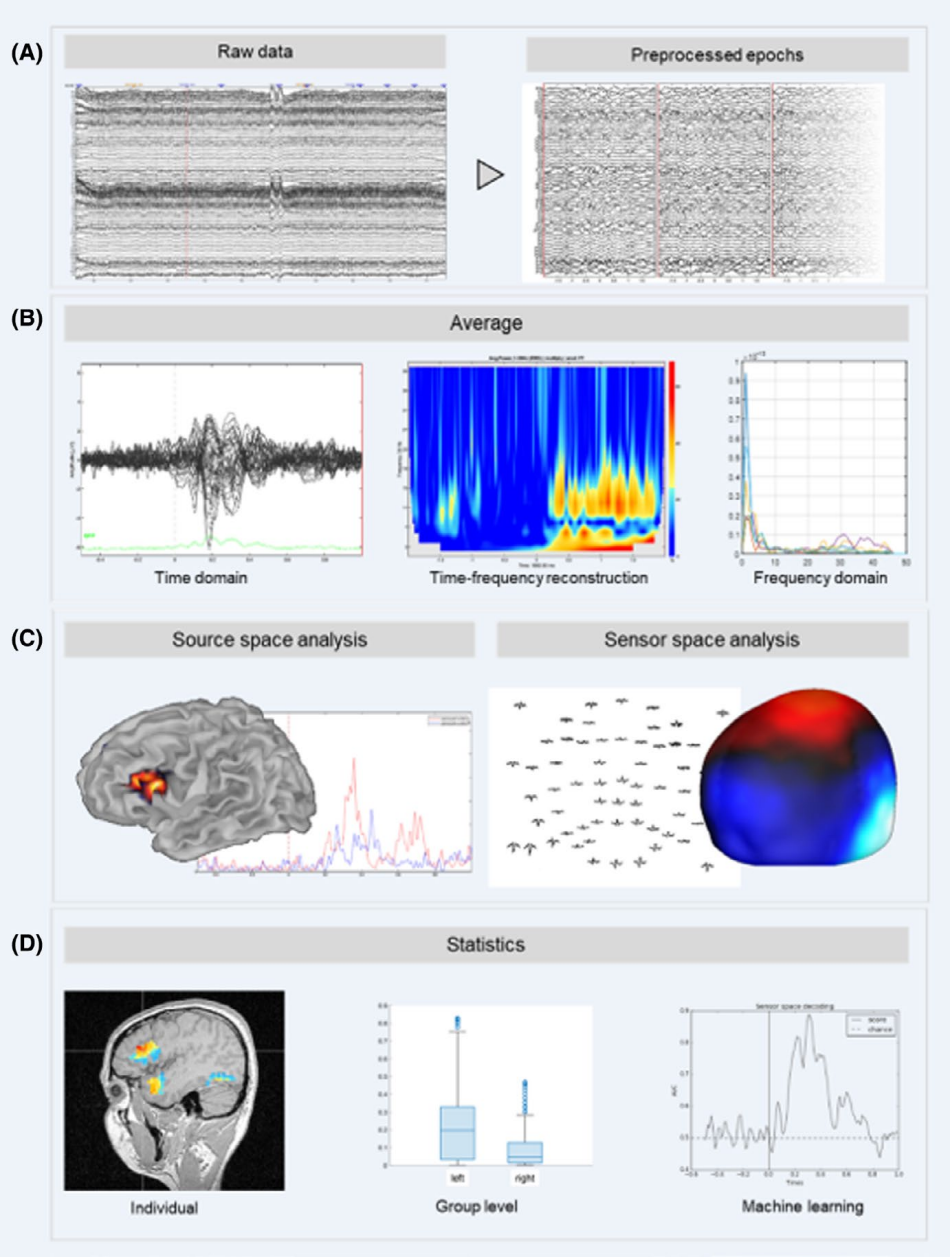
An overview of typical EEG processing and analysis. (A) Recorded raw data are preprocessed to reduce artifacts (e.g., power line noise, EMG activity, eye blinks, etc.), exclude noisy channels and segments and divide the data into short epochs. (B) Epochs are averaged in the time or frequency domain. Alternatively, epochs are first converted to time–frequency reconstructions before averaging. (C) Data are analyzed in sensor (electrode) space, that is, time series of the different channels to evaluate topography and extract any features of interest. Alternatively, the data are submitted to source imaging to project the electrode time series into source space before evaluation of spatial distribution and feature extraction. (D) Features of interest are then evaluated on an individual level, for example, for clinical applications, or analyzed using group level statistics and machine learning approaches.

Standard preprocessing^18^ consists of frequency filters to reduce low‐ and high‐frequency and powerline noise (Figure 3). Clinically, interpretation is performed by visual inspection of morphology and topography and utilizing montages that permit interpretation through reference schemes of the recorded data. Conventional EEG markers of epilepsy are interictal epileptiform discharges (including spike‐/sharp‐wave patterns, pathological high‐frequency oscillations), ictal rhythmic seizure patterns, and alterations of the normal physiological oscillatory background, such as focal or generalized slowing.^17^, ^19^ Advanced methods require additional preprocessing techniques, such as principal and independent component analysis, source localization, and quantitative/statistical methods.^18^ Source localization techniques (“source analysis” or “source imaging”) project the surface data into the anatomy, partially compensating for the influence of volume conduction, source orientation, and simultaneous activation of multiple sources. Quantitative methods describe statistical properties of either raw or source‐localized data, ranging from power in specific frequency bands, the degree of randomness, occurrence, and stability of microstates, to synchronization and functional connectivity between EEG channels, locations, and signal components (cross‐frequency coupling). Further methods enable the exploration of directional connectivity and aperiodic, non‐oscillatory components. Efforts to leverage large‐scale EEG analytics, though, have suffered from low statistical power secondary to limited sample sizes. This problem is multifaceted but relates in part to “siloed” and inaccessible data, proprietary software with limited interoperability, and, for intracranial EEG, lack of standardized implantation schemes. However, international collaborations availing themselves of standardized methods of data collection now exist and have yielded promising initial returns reflecting the value of AI in automated EEG interpretation when evaluating over 30 000 recordings.^20^

#### 2.1.3 MRI

**FIGURE 4:**
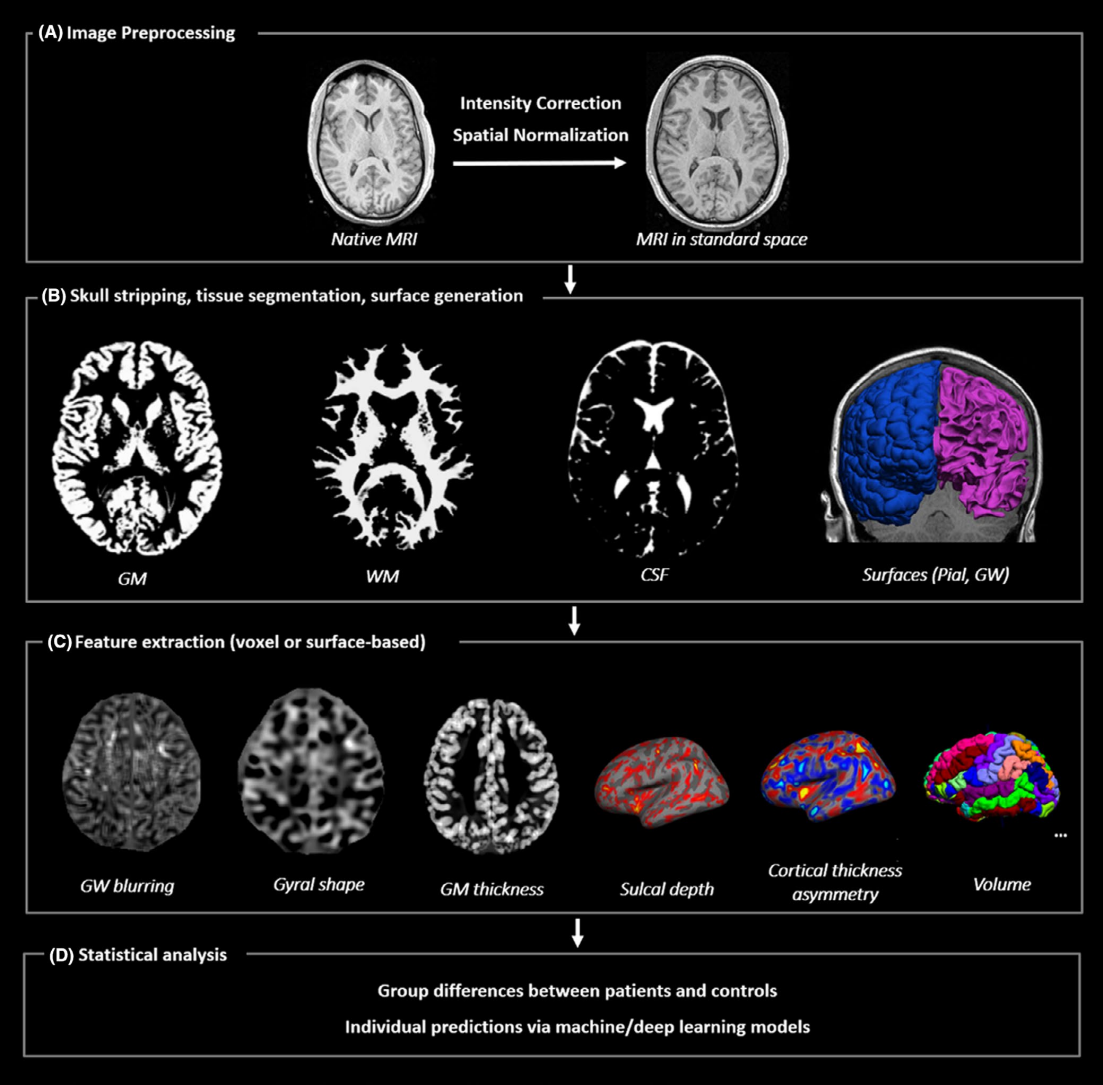
An overview of typical MRI processing and analysis. (A) Raw MRI data are preprocessed to reduce artifacts (e.g., field inhomogeneity) and spatially normalized to standard space. (B) Skull is then removed from the image volume, followed by tissue segmentation which allows for separation of brain tissue to GM, WM and CSF. Pial or GW surfaces can also be generated. (C) Feature extraction based on the MR images can be performed on voxel level or surface level. Common features include GW boundary blurring, gyral shape, GM thickness, sulcal depth, interhemispheric asymmetry measures, volume, etc. The choice of feature depends on specific goals of each study. (D) Features of interest can then be statistically evaluated to identify group‐level differences or perform individual‐level predictions.

An MRI is a critical investigation for people with epilepsy as it permits the identification of a potentially epileptogenic lesion and its surrounding anatomy.^21^ A typical MRI epilepsy protocol usually includes a 3D T1‐weighted volumetric acquisition, coronal thin‐section T2‐weighted and fluid‐attenuation inversion recovery (FLAIR) acquisitions. Of these, the 3D T1‐weighted volumetric sequences provide exquisite anatomical detail from contiguous thin slices (with no inter‐slice gap) that can be reformatted to any plane; therefore, these sequences are usually used for image processing studies. As shown in Figure 4, an essential first step for most image processing methods is the correction of MR field inhomogeneities, which cause variation of signal intensity across the brain. The next step is the alignment of images to a 3D standard space, which enables the comparison of brain structures among different individuals. Then, each voxel is classified into tissue classes, namely gray matter, white matter, and cerebrospinal fluid. Tissue classification and segmentation provide the basis for further voxel‐based or surface‐based analyses on the group level or individual level. While the clinical role of MRI for epilepsies is well established,^22^, ^23^ most published research studies involving brain morphology and functional connectivity have had limited power due to the small number of participants.^24^ Similar to emerging large‐scale EEG studies, several international collaborations have facilitated data collection and standardized analyses to generate impactful research, including determining the prevalence of structural abnormalities in epilepsy^25^ and their geographic variation in relatively large cohorts, and thus are leading efforts to foster Big Data MRI epilepsy research.

#### 2.1.4 Neuropsychology

The epilepsy neuropsychology community has yet to reach international consensus regarding “gold standard” tests, critical cognitive domains, and definitions of impairment, which has previously hampered Big Data efforts. However, global harmonization is a priority of the ILAE Neuropsychology Task Force, which has developed the International Classification of Cognitive Disorders in Epilepsy (IC‐CoDE) to address this issue.^26^ The IC‐CoDE includes international consensus‐based cognitive domains, test exemplars, and diagnostic algorithms to derive cognitive phenotypes in epilepsy, which can be applied at the individual level and across cohorts. This approach is flexible and has been tested in English‐ and Spanish‐speaking adults with focal epilepsy in the U.S.^27^, ^28^, ^29^ Efforts are now underway to apply the IC‐CoDE to culturally and linguistically diverse populations internationally, thus moving toward Big Data research.

#### 2.1.5 Genetics/omics

The epilepsy research community has identified over 900 genes that cause monogenic forms of epilepsy^30^ and are steadily unraveling the genetic architecture of other multifactorial forms of epilepsy.^31^, ^32^ Genomic tests such as array comparative genomic hybridization and exome/genome sequencing have become routine epilepsy clinical tests.^33^ Somatic mutational analysis has recently been implemented for a spectrum of malformations of cortical development.^34^, ^35^ Genomic data for the epilepsies are available from both research and clinical sequencing. Although there are notable efforts to centralize datasets and coordinate analysis (such as the Epi25 and EGI initiatives),^36^ much of the data remains siloed, particularly in the clinical setting. Moreover, due to variations in the degree of clinical phenotype information, such as the ILAE epilepsy type and semiology, it is necessary to consider quality control and the interoperability of the formats in which these characteristics are defined and stored. In response to this challenge, the ILAE Genetics Commission has initiated “ILAE Genomics,” designed to assemble and standardize genetic and phenotypic datasets of people with epilepsy worldwide and make them available to the research community (https://www.ilae.org/about‐ilae/structure‐and‐working‐groups/commissions‐and‐sections/commission‐on‐genetics). A long‐term goal is to identify, centralize and make these datasets interoperable which will in turn enable rapid and cost‐effective progress for continued translation genetics research in the epilepsies.

#### 2.1.6 Wearables and person‐derived digital information

Commercially available fitness wearables and smartwatches record a huge amount of biosignal data, including accelerometry, electromyography, electrodermal activity, and heart rate.^37^ Several wearable devices have been developed and validated for the detection of epileptic seizures using inpatient video‐EEG monitoring as a gold standard.^38^ Most of these devices detect generalized tonic‐clonic seizures (including focal‐to‐bilateral and generalized onset tonic‐clonic seizures) with a high sensitivity (over 90%) and a reasonable false alarm rate (0.2–0.7 / 24 hours).^38^ Absence seizures can be detected using dry EEG electrodes,^39^ and promising results have been published about the detection of non‐convulsive seizures using heart‐rate variability.^40^ Convulsive functional seizures are also being identified using accelerometers, reporting a mean sensitivity of 62.9% and a false alarm rate of 0.8 / 24 hours.^41^

Direct person‐derived information is valuable. Mobile smartphone applications now allow people to directly document seizure frequency, intensity, triggers, medication adherence, and adverse events. These data can also be used to derive real‐world evidence about epilepsy, though caution due to underreporting is recommended.^42^ Implantable devices, such as responsive neurostimulation, may permit more granular longitudinal research‐ready data on seizure frequency.^43^

However, most of these devices (and the data they collect) are proprietary, which precludes their use for the general benefit of people with epilepsy. There is a need to persuading the manufacturers that, after appropriate consent, de‐identified data collected using wearables should be made available for research and development. Further, these devices are not always tailored for people with epilepsy, whose acceptance of wearable technology for seizure detection is strongly influenced by accuracy, design, comfort, and cost.^44^

### 2.2 A team‐based approach to improving the epilepsy landscape

**FIGURE 5:**
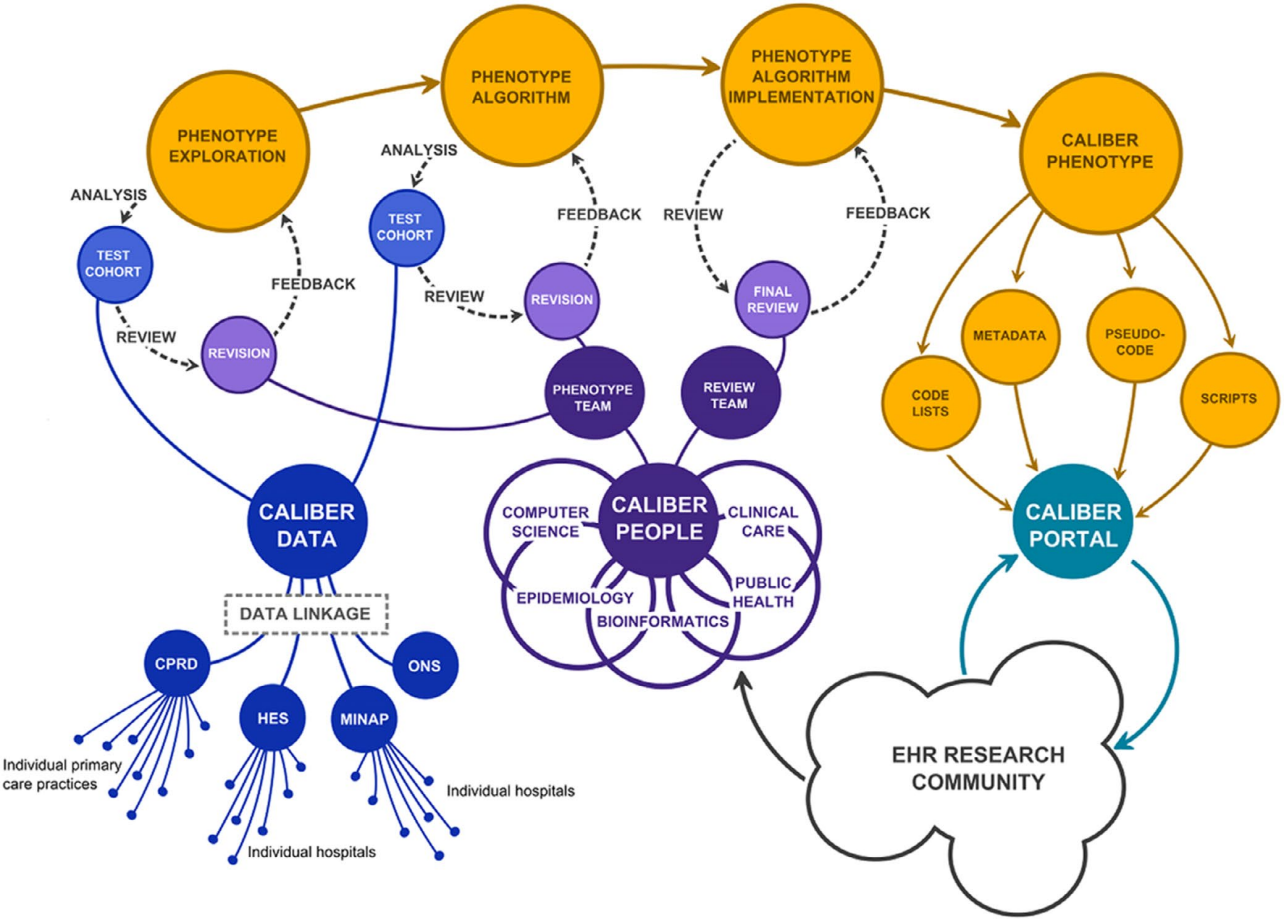
Phenotype generation is critical to accurate and reliable Big Data studies employing electronic or administrative health records. A systematic approach involves first exploring the data to find relevant codes and concepts, then using these data to develop and test algorithms meant to isolate people with the disease of interest. The algorithms can then be deployed to test their accuracy using real world data. Finally, once refined and optimized, these algorithms can be stored in centralized repositories where they can be readily deployed for research and surveillance purposes. Reproduced from 49. CPRD represents the Clinical Practice Research Data link; HES represents Hospital Episode Statistics; MINAP is the Myocardial Ischaemia National Audit Registry; ONS is the UK Office of National Statistics (mortality and social deprivation data).

Clinicians skeptical about what Big Data can provide are often worried about the concept of “garbage in, garbage out.” The misdiagnosis rate of epilepsy can be as high as 20%,^45^ and that for seizures potentially even higher, meaning that just because the person has a diagnostic code for epilepsy does not guarantee that they have the disease.^46^ Creation of valid, reliable, and transferable case definitions/phenotypes of epilepsy is thus essential (Figure 5).

To this end, it is integral to educate all health care professionals and people with epilepsy about the emerging role Big Data will play in their lives. Charting is not just important for clinical safety; its downstream effects also influence “real‐world evidence” research. Thus, accuracy is paramount if we truly intend to glean novel insights from real‐world evidence and learn about health systems. Physicians and nurses should aim to use specific epilepsy codes that reflect ILAE classification schemes in everyday practice to capture complexity and heterogeneity. Updating problem lists and comorbid diseases facilitates Big Data research that attempts to understand epilepsy holistically. Detailed notes from allied health professionals can be parsed through sophisticated techniques such as natural language processing to supplement structured and semi‐structured data.

Health care providers can be incentivized to improve Big Data in practical, everyday settings if they are able to readily access efficient and effective electronic communication, reminders, data visualization, and decision‐support tools that save time and augment care.^47^ People with epilepsy should be empowered to actively and safely contribute data and, with the advent of accessible personal records, be encouraged to review their clinic notes and request amendments when inaccuracies are encountered. They should also be empowered to advocate for safe, transparent, ethical, and equitable processes that optimally balance privacy concerns with the innovative and transformative potential of Big Data research.

### 2.3 Linkage and application to AI

#### 2.3.1 Linkage schemes

To truly harness the power of these individual sources of epilepsy data, intermediary steps are required to amalgamate them into a single high‐dimensional analytics‐ready dataset. Deterministic and probabilistic linkage schemes can be deployed to accomplish this. Deterministic linkage involves creating rules that identify and link data points from discrete datasets that belong to the same person. For instance, this would require exact agreement on personal identifiers, such as a person's health number, date of birth, sex, and address,^48^, ^49^ which can be challenging due to privacy concerns. Although inadvertent false positive linkages can be made, this is rare given the specificity of these identifiers. When unique identifiers do not exist, probabilistic linkage offers a more flexible approach, using a wider range of variables, such as name, region of residence, and age, to link records dependent on the probability that they relate to the same person.^48^ The benefit is that deidentified or incompletely identified data can be linked using a series of non‐uniquely identifying variables, thus increasing sensitivity, though the negative effects on specificity should be judiciously considered.

#### 2.3.2 AI

**FIGURE 6:**
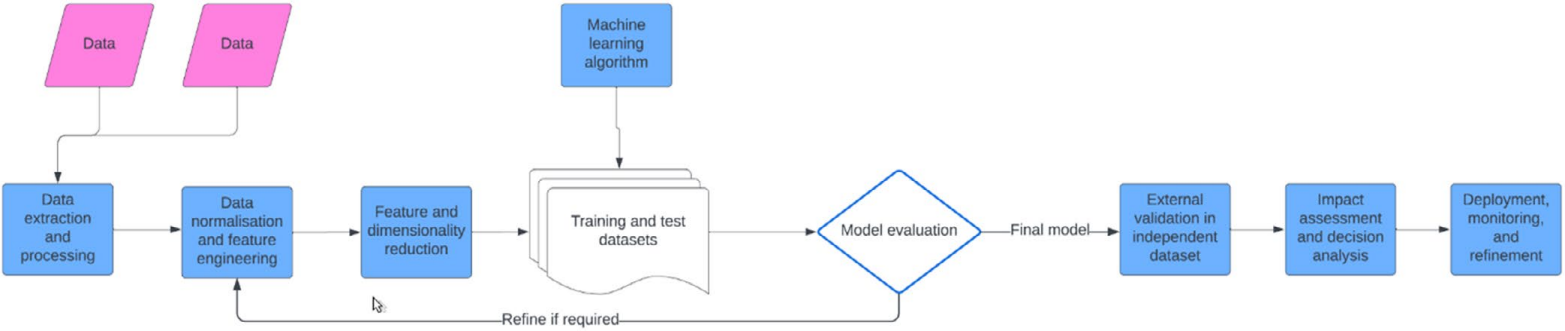
The general approach to generate machine learning and artificial intelligence models. Data can be obtained from a single or multiple datasets. The data are then extracted, normalized, and processed to permit feature (independent variable) identification. These features may need to be engineered from the raw data. Feature and dimensionality reduction is then performed to reduce the risks of overfitting and modeling in sparse dimensional space. The final analytics dataset is then divided into training (where the model is derived) and testing (where the model is tested, and performance metrics are derived). If the discrimination, calibration, and overall performance metrics are suboptimal, then attempts can be made to refine the model by re‐engineering and re‐selecting features. Once the final model is derived and decided upon, it should then undergo external validation in an independent population to ensure performance is generalizable. If it is, and still performs well, then further impact assessments and decision analyses are required to ensure it truly enhances care, is feasible in real world settings, and does not adversely affect health systems. Following this, if these measures are met, the model can finally be deployed for use in care and disease monitoring, and the algorithm can be perpetually refined in a continual learning environment as it receives novel real‐world data in electronic health records systems.

A comprehensive overview of AI is beyond the scope of this article, but general approaches exist for processing and analyzing data (Figure 6). Briefly, once the linkage is complete, the final analytic dataset is created. However, before one can model the data, crucial pre‐processing steps may be required, including data cleaning (which involves, but is not limited to, coding data in analyzable formats, removing duplicate or irrelevant observations, and addressing missing data), feature engineering, and dimensionality reduction. Data are rarely complete, and decisions need to be made regarding performing a complete case analysis or, where possible, to avoid bias and imputing values.^50^, ^51^ Likewise, AI models are at risk of running afoul of the “curse of dimensionality.” This occurs when an excessive number of features (independent variables) spanning many dimensions (axes on a graph mapping the data) are deployed in models applied to small study populations. This can lead to performance issues, multicollinearity, overfitting, and multiple testing.^52^ Hence, although arguably less important for specific forms of AI, such as deep learning and generative AI, feature engineering—broadly encompassing feature selection and dimensionality reduction—remains a critical pre‐processing step. By synthesizing new variables through combining and/or re‐processing the original raw features, more informative ones can be created. This is arguably particularly useful for epilepsy research since complex MRI images, EEG signals, and genomic data are not easily expressed as simple and interpretable “independent variables.” Thus, feature engineering can not only improve algorithm performance, enhance internal and external validity, and importantly, avoid producing fallacious results that can result from modeling noise or corrupted data,^53^ but should also specifically facilitate the application of AI to epilepsy. The drawback, though, is that engineered features and “black box” AI models (AI systems that use opaque mechanisms to process variables and reach decisions) are challenging to interpret, rendering auditability, verification, and understanding of individual decisions problematic, if not impossible.^54^ Such models may be particularly prominent in epilepsy if the models are highly reliant on complex EEG, MRI, genetics, and wearable data. The trust health care providers have in AI is directly related to their ability to explain and comprehend how the models reach specific decisions,^55^ and therefore it will be important to prioritize explainable AI where feasible.

## 3 ETHICS OF BIG DATA

### 3.1 Informed consent

While accumulation, processing, and analysis of Big Data can now be accomplished using the steps described above, there remain many evolving ethical issues. Free, informed, voluntary, and enduring consent is a crucial part of medical research, as stipulated in the Nuremberg code and Declaration of Helsinki. These regulations are easily applied to conventional prospective studies. However, the informed consent process has become murky in the era of Big Data. Big Data research typically constitutes secondary data use, with researchers rarely involved in collecting the primary data or directly consenting participants.

In the United States, studies using anonymized and aggregated data are governed by the “broad consent” clause of the Common Rule, and the Health Insurance Portability and Accountability Act (HIPAA) does not place restrictions on the use of deidentified data for research purposes.^56^, ^57^ Similarly, in the European Union, research on irreversibly anonymized data is exempt from the General Data Protection Regulation (GDPR).^56^ The assumption is that risks are minimal if the data cannot be tracked back to the original person. However, with increasing sophistication of AI and computing power, certain individuals can be re‐identified despite anonymization.^58^ Thus, novel approaches to increase awareness of these issues are urgently needed. These may involve safeguards such as tagging data so people can see how they are being used, regularly consulting with marginalized and underprivileged groups to ensure cultural and personal safety, and continually reviewing and engaging with all affected parties about evolving de‐identification techniques and governance mechanisms to ensure we can maximize the potential of Big Data without jeopardizing autonomy, trust, and safety.^56^, ^57^

### 3.2 Data ownership, privacy, and access

Another ethical dilemma is data ownership, especially with respect to wearable devices. People who are contributing data quite rightly feel entitled to agency over their information and how it is used. The problem lies in identifying what type of ownership this constitutes (is it a private or public good) and whether people providing their data can or should commercialize access. Enabling individuals to share their data as a private financial commodity could impede research efforts by introducing selection bias and/or rendering studies cost‐prohibitive. Routinely providing individual‐level compensation, at its very least, could represent a serious bureaucratic hurdle and, at its worst, risk exploitation by intermediary corporate health databanks. Conversely, making such data broadly accessible as a common good raises the issue of who will bear the brunt of the organizational and procedural costs and who ultimately is responsible for ensuring privacy and regulating access.^59^ Data Access Committees (DACs) are critical to balancing these competing interests and rights. They can help ensure that privacy and confidentiality are protected, that incentives are made available for primary data collection and research, and directly provision sound governance frameworks. These DACs should be composed of data science and ethics professionals, as well as people with epilepsy and their advocates, who act as custodians as opposed to proprietary owners.^59^ Finally, critical to epilepsy research, where the disease is more prevalent in low‐ and middle‐income nations, DACs should ensure fair and equitable access, as well as financial and technical support, since evidence exists that resource‐challenged settings are at a disadvantage when attempting to access secondary data.^60^

### 3.3 Data bias and algorithm objectivity

Issues of bias remain paramount when considering the use of AI. The problem can be dissected into issues of prediction and decision allocation. Specifically, prediction refers to the accuracy of the AI, while allocation refers to how one makes a decision based on the AI's output.^61^ Due to bias in the underlying data, some algorithms can be “prejudiced,” lacking accuracy or displaying systematic bias against specific groups of people. This is concerning in epilepsy, where disparities in access to diagnosis and treatment persist for minority and underprivileged groups.^62^ This can lead to systematic errors when trying to predict outcomes in underrepresented groups. Likewise, algorithms can be prejudiced due to design choices—inclusion or exclusion of specific features, application of certain privacy‐preserving techniques in imaging analyses (such as defacing MRIs), and data compression can introduce bias against specific subgroups.^63^

On the other hand, the algorithms may not be biased with respect to prediction but can lead to outputs that promote inequitable and unfair decision allocation and fail to maximize utility. For instance, if the algorithm is designed to predict injury risk and assigns a lower value to those who are seizure‐free, employers could use this to reject gainful employment to people with drug‐resistant epilepsy, thus exacerbating disparities. A more equitable outcome would be to offer job accommodations to ensure safety at work rather than using algorithms to myopically guide employment decisions.^61^

In summary, the societal implications are large. Researchers, health care providers, and people with epilepsy are left with two choices. The first is to wait passively until society changes, hoping more equitable data becomes available. The second is to use health informatics methods to identify data and algorithmic bias and then decide on how to use this information to deliberately promote societal change (and routine data collection) in a positive way. Ultimately, a proactive approach is needed to yield ethical frameworks that ensure equitable allocation of AI‐based prediction decisions. Such efforts are now being pursued, as formal governance processes are being created to garner trust among the public and health care providers. A welcome step is the 2023 Bletchley Park Declaration, comprising 28 nations and the European Union, which has formally stipulated that global action is needed to tackle risks, such as promoting inclusive measures to ensure ethical, trustworthy, and safe AI is achieved through appropriate levels of government regulation.^64^

## 4 COMMUNITY ENGAGEMENT

### 4.1 Why is it Important?

Engaging people affected by epilepsy and all other parties with a direct stake in the condition is critical for the planning, conduct, and dissemination of research projects.^65^ The international epilepsy community is a pioneer of engagement, with some of the earliest advocacy groups in the world. People with epilepsy and caregivers offer valuable perspectives in research preparation, execution, and translation.^66^ They provide the lived experience perspective, which can help highlight research priorities and identify person‐centered outcomes. Their critical efforts include contributing to Core Outcome Set development,^67^ supporting recruitment and retention in longitudinal studies, advising on ethical issues, and disseminating study results beyond the scientific community.^68^ Advocates also work toward improved outcomes for rare epilepsies,^69^ regulatory approvals,^70^ collaborations on research, including registries,^71^, ^72^, ^73^ and providing advice on the development of clinical guidelines.^74^ In the context of Big Data and AI, a recent qualitative study found that people with epilepsy were cautiously open to AI models being used as clinical decision support systems but highlighted that endorsement by their neurologist was important to accepting these models.^75^

### 4.2 How do we do it?

Many excellent frameworks exist for soliciting participation and mapping a participant's journey in research, including Big Data^76^, ^77^, ^78^, ^79^; but there are few examples in the literature of data science activities actually availing themselves of these tools to achieve high levels of community engagement.^66^, ^80^ People affected by epilepsy and advocacy groups should be invited to contribute early to all phases of the research process. People with lived experience should be equal partners in academic endeavors and conferences, separate from their role as a person with epilepsy or a caregiver, aiming for the co‐production of research. Such engagement should be planned with local research ethics boards to avoid conflict of interest and ensure compliance with regulatory policies such as HIPAA and GDPR.

## 5 THE ENVIRONMENTAL IMPACT OF BIG DATA AND AI

An increasingly recognized cost of the inevitable generation, movement, and manipulation of Big Data and its applications is its carbon footprint. The climate is changing due to human activity—2023 was the hottest year on record, with records broken across the world.^81^ The health impacts of these changes are rightly receiving increasing attention, with joint editorials across leading medical journals in 2021^82^ and 2023^83^ calling for urgent action. Healthcare accounts for ~4% of greenhouse gas emissions globally,^84^ and two major contributors to Big Data efforts are significant: medical imaging and genomic data, both of which are commonly used in epilepsy. Across 120 countries, MRI and CT scanning produces an estimated 0.77% of global greenhouse gas emissions.^85^ The emissions per MRI scan depend on many factors, and the overall energy consumption varies with machine utilization, but are of the order of 17.5 kgCO_2_equivalent/scan, roughly equivalent to driving a new car 175 km^86^. Genomic data analyses, including the running of algorithms, have their own carbon footprints.^87^, ^88^, ^89^ Newer Big Data efforts, such as analyses of phenotype data and the implementation of AI techniques^90^, ^91^ will have significant associated carbon costs. Data storage and transfer are also important contributors: estimates are that data centers use more energy than some entire nations and collectively account for 0.3% of global carbon emissions.

Big Data holds much promise, but its implementation must take these carbon costs into account: as scientists basing practice on evidence, we have to acknowledge that our own work has an environmental cost, and we must seek to reduce that cost, not least because climate change, to which we are contributing, is likely to directly affect people with epilepsy, as implicit in the establishment by the ILAE of the Climate Change Commission. With regard to Big Data, we can reduce our impact by how we generate data,^86^, ^92^ and store, use, and disseminate it^88^. As climate change takes hold, there will be threats directly to Big Data efforts, as heatwaves compromise computing facilities.^93^, ^94^, ^95^ We must, therefore consciously anticipate these changes and plan now to be more sustainable and resilient in our work.

## 6 BARRIERS TO UPTAKE OF ALGORITHMIC BIG DATA RESEARCH

### 6.1 Internal and external validation, real‐world performance and validation, and governance

Current precision and personalized medicine models often poorly report or suffer from methodological shortcomings that impact validation and reliability.^51^ Recommendations have been proposed to enhance the internal validity (the degree to which the study results represent the true effect in the source population) of models generated from clinical data.^51^, ^96^ Once internal validity is established (by avoiding systematic bias and accounting for confounding), we can confidently assess the model using measures of discrimination (its ability to distinguish between individuals with and without the endpoint of interest), calibration (a comparison of the observed and predicted risks), and overall performance. The majority of published models end the process here,^96^ or are subject to poorly described or insufficient external validation studies.^97^ This is a critical barrier since performance almost invariably drops after external validation by independent investigators.^97^, ^98^ Published recommendations to evaluate measures of external validity^96^ should be pursued for all models before considering deployment in real world settings.

Following external validation, decision curve analyses and studies, ideally randomized controlled trials, that evaluate health systems performance are critical next steps. Decision curve analyses go beyond discrimination and calibration by conveying the clinical utility or “net benefit” of a validated algorithm compared to routine clinical practice.^99^ In essence, this balances preference (threshold probability) for a test with net benefit. If a test is low risk (e.g., EEG) and/or the outcome is severe (e.g., status epilepticus), a model may not be required to support the decision to order a test. Conversely, if a test is of higher risk (intracranial EEG) or the outcome is of a lower severity or unlikely (seizure freedom in multifocal epilepsy), a model may again not be required since one would typically avoid the test. Ultimately, a model conveys value if it leads to greater net benefit in situations where the risks and benefits of a test and particular outcome are closely balanced.^100^

Real‐world performance must also be formally evaluated. Highly accurate AI algorithms exist that paradoxically disrupt care when deployed in settings that lack the resources and computing power to run them.^101^, ^102^ Thus, trials reporting individual outcomes and health systems impacts are crucially needed prior to deployment. After a fully validated model with proven real‐world benefit is implemented in clinical practice, ongoing assessments of its performance are necessary as its data sources may evolve with changing architectures of the EHR or clinical practice workflows.

### 6.2 Infrastructure, computing power, and proprietary issues

Assuming internally and externally validated algorithms are created and are demonstrated to have a meaningful impact on individual and health systems outcomes, subsequent barriers include local infrastructure and workflow integration. Ideally, the results of Big Data research are flexibly incorporated in the EHRs, and those that have had success provide decision support, i.e., automatically incorporated into clinical workflow, provide advice at the time and location of decision‐making, and provide actionable recommendations.^103^ Recommendations and quality improvement programs exist that guide development and encourage judicious use of decision support tools, thus facilitating their use in these environments.^104^, ^105^

Given these are the results of Big Data algorithms, this presupposes the presence of intuitive interfaces embedded in a high‐performance computing environment with adequate bandwidth to provide expeditious output.^106^ In low‐ and middle‐income countries where access to substantial resources might be limited, Big Data analytics can be challenging to implement. This potentially creates a reality in which high‐income countries disproportionately reap the benefits of sophisticated AI whilst low‐ and middle‐income countries struggle to span the divide due to a lack of internet bandwidth, computing power, advanced graphics processing units, and skilled personnel.^107^ Thus, as a community, we need to actively advocate for improved internet bandwidth and access to cloud computing where environmentally sustainable. We must also engage computer science and information technologists to generate open‐source products and analytics libraries. Relational data stores can be implemented using MariaDB, and analytics tools can be developed using open‐source libraries such as Scikit learn (Python) and R. International standards, such as ICD‐10 and SNOMED‐CT, can be used to standardize data and, where appropriate, open‐source CDMs such as OMOP can be deployed to create harmonized data pools.

Deploying such open source solutions can be problematic when proprietary ownership leads to limited flexibility and results in cost‐prohibitive barriers that deter data sharing and implementing prototype models.^108^ Government interventions that ensure proprietary data formats are converted to CDMs and made available on commercially accessible and integrated cloud storage have been proposed as mechanisms for enhancing innovation and promoting public‐private partnerships that accelerate meaningful Big Data research.^108^

## 7 EXEMPLARS OF BIG DATA AND ADVANCED ANALYTICS

Despite these challenges, considerable progress has been made in producing impactful Big Data research in epilepsy. The SCORE‐EEG system (Standardized Computer‐based Organized Reporting of EEG) is a software tool for standardized EEG annotation that automatically generates clinical reports and feeds the data (extracted features) into a standardized database.^109^, ^110^ Using a convolutional neural network, a model (SCORE‐AI) was developed using more than 30 000 de‐identified EEGs from two major centers.^20^ The model was trained to separate normal from abnormal EEG recordings and then classify abnormal recordings into categories relevant for clinical decision‐making: focal epileptiform, generalized epileptiform, focal non‐epileptiform, and diffuse non‐epileptiform abnormalities. Afterward, the model was tested on three independent datasets constituting nearly 10 000 EEGs derived from external centers. SCORE‐AI achieved performance similar to human experts in the automated interpretation of routine clinical EEGs.^20^ The key to the success was the availability of large EEG datasets with standardized annotations.

Similarly, recent large‐scale, worldwide collaborative projects such as ENIGMA‐Epilepsy^111^ and Multi‐centre Epilepsy Lesion Detection (MELD)^112^ have further demonstrated the importance of multi‐center collation of epilepsy data. ENIGMA‐Epilepsy have analyzed over 2000 MRIs and confirmed widespread gray matter atrophy^113^ and white matter changes,^114^ providing unique information on common and distinctive structural differences among different epilepsy syndromes unlikely to be detected by single‐center cohort studies. The MELD project used machine‐learning methods to develop an algorithm for the automated detection of focal cortical dysplasia. Deep learning approaches have also shown promise in a recent multi‐center study, where MRI data were used to train a deep convolutional neural network (deepFCD).^115^ New tools like these may assist physicians in recognizing subtle MRI lesions and offer surgery for some people with a previous diagnosis of “normal” or “negative MRI.”

## 8 THE ROAD AHEAD—WHAT IS THE FUTURE OF BIG DATA IN EPILEPSY

We are now firmly in the age of Big Data and AI. This means as a community, epilepsy health care providers, researchers, and people with epilepsy must educate and prepare themselves for the impact this will have on their lives. To avail ourselves of the benefits at an individual and population level, we must urgently learn to homogenize and share or federate data, set standards and regulations for data integration and analysis, and engage with all people affected by epilepsy to identify immediate priorities. People with epilepsy remain at risk of stigma. Thus, it is crucial we do not allow Big Data and AI to exacerbate existing global socioeconomic and climate disparities. It is equally crucial we ensure personal agency with respect to consent and data ownership. To accomplish this, a shift from artificial to decision intelligence may be required. Decision intelligence involves convening health informaticists, data engineers, health care providers, ethicists, and people with epilepsy at an early stage to establish what is important, needed, and feasible and to proactively identify barriers and opportunities for ethical AI.^116^ By understanding the underlying mechanisms and utilities used to make decisions, we can prioritize practical and high yield needs to ensure maximal returns in fragmented and often unequal health care systems.

**Glossary**
Administrative health records:
these are documented records generated during interactions with a health care system. These include visits to physician's offices, emergency department visits, admissions to hospital, prescription fills, receipt of laboratory or diagnostic imaging, and vital statistics such as births and deaths.
Algorithmic bias (prediction bias):
a systematic (not random) and persistent error that results in inaccurate or unfair outcomes. This can result from biased input data or inadvertently prejudiced design or deployment of a predictive model.
Artificial intelligence:
intelligence exhibited by machines or the ability of a digital program to perform tasks and solve problems in ways commonly associated with intelligent beings.
Big Data:
conventionally defined as data that are too large or complex to be analyzed by conventional data‐processing approaches. It is often defined by a series of ‘v's that include ‘volume’ of data, ‘variety’ of the data, ‘velocity’ of data production, ‘veracity’ of the data, and ‘value’ of the data.
Bletchley Park Declaration:
A declaration signed by 28 countries agreeing to promote global efforts to leverage the benefits of AI in a safe, equitable, ethical, and effective manner.
Calibration:
is the accuracy of absolute risk estimates. It is the degree to which the predicted risk probability aligns with the observed proportions that experienced the outcome of interest.
Case definition/phenotype:
algorithms that employ disease and symptom classification coding terms and diagnostic tests to identify people with a specific disease of interest in electronic and administrative health records data.
Cloud computing and storage:
on‐demand availability of computer system resources, including computing power and storage. These are distributed across geographically diverse data centres to reduce expense and share resources to optimize performance.
Common data models:
schema that promote harmonization of disparate data sources by mandating standard definitions and structure using *a priori* semantics, organization, and analysis methods.
Convolutional neural network:
a type of artificial intelligence that automatically extracts and engineers features (independent variables) and then passes them through multiple analytic layers to classify outcomes. It is particularly useful for image‐recognition tasks.
Core outcome sets:
an agreed upon minimum, standardized set of outcomes that should be measured and reported in all clinical studies for a specific disease or area of health care.
Curse of dimensionality:
poor model performance that arises when too many variables are applied to too few data. This results because each variable represents a dimension (an axis of a graph) in analytical space. When datapoints (study subjects) are mapped through an increasingly larger number of dimensions, the space between them is greater which leads to poor performance when models use distance as a measure of similarity.
Decision allocation bias:
when a model is accurate and free of algorithmic bias, but its recommended decision conflicts with the principles of distributive justice (the fair and equitable allocation of resources, goods, and opportunity in a society).
Decision curve analysis:
an analytic method that calculates a clinical “net benefit” for a model. It compares the relative benefit of using a prediction model at specific threshold preferences to the default strategies of either intervening on everyone or intervening on no one.
Decision intelligence:
a discipline that advances decision making by explicitly understanding and engineering how decisions are made and how outcomes are evaluated, managed and improved via early engagement and iterative feedback from all affected parties.
Deterministic linkage:
is a method that links data for the same person from different datasets. This approach uses a series of decision rules to determine whether the data from each dataset pertains to the same person. An example is combining rows from two separate datasets using a key variable, such as a personal health identifier.
Dimensionality reduction:
transformation of data from a high‐dimensional (high number of features) dataset into a lower dimensional (fewer features) dataset whilst still retaining meaningful properties from the original data. This can be accomplished by applying algorithms that amalgamate or process a set of original variables into a single summary variable that can be deployed in the analytic model in place of the original variables.
Electronic health records:
a digital version of a person's chart. This contains longitudinal information on a person's history of disease, tests, and treatments that provides an all‐encompassing overview of the individual's health.
External validity:
results are externally valid when causal relationships and statistical associations determined from an initial study can be replicated and applied to different populations, settings, and times.
Fast Healthcare Interoperability Resources (FHIR):
is a Health Level Seven International® (HL7®; a not‐for‐profit, American National Standards Institute‐accredited standards developing organization) set of rules and specifications for facilitating the electronic exchange of healthcare information through interoperability of electronic health records systems.
Feature engineering:
a preprocessing step in machine learning whereby raw data are transformed into a more effective set of model inputs. For instance, using comorbidity indices or calculating interaction terms can be ways of reformatting raw data to facilitate analysis and improve model performance.
Feature selection:
methods by which an analyst can select a series of features (variables) to identify the optimal subset that conveys the strongest predictive power.
Federated data analysis:
a method of de‐centralized data analysis. Using federated learning, a pre‐trained foundation model is developed and then sent to local sites. Each site then trains the algorithm on their own local private data, and then summarizes and encrypts their own version of the model which is sent back to a central organizing site. Here, each site's model is decrypted, averaged, and integrated into the foundation model. This process occurs iteratively until a final model is created. Data therefore remain private and secure as they are never shared or transferred outside the local collaborating centre.
General Data Protection Regulation (GDPR):
a European Union law that mandates how organizations and companies must use personal data. It provides a legal framework for data protection and privacy for all individuals within the EU, including how physical or legal persons may use and process personal data.
High dimensional data:
datasets in which the number of variables is larger than the number of observations. The term high dimensional derives from the fact that each variable represents a dimension (an axis of a graph) in analytical space. Usually, data are considered ‘high dimensional’ when their number is higher than that usually used in conventional biostatistical analyses.
Health Insurance Portability and Accountability Act (HIPAA):
a federal act that governs health insurance, transactions of electronic health information, and data protection in the United States.
Independent component analysis:
a computational method used in signal processing that aims to identify and separate the additive independent sources that comprise signal data.
Internal validity:
the extent to which the study results represent the true population effect size. This can be determined by evaluating the study's design, conduct, and method of statistical analysis.
Model discrimination:
this is a measure of how well a model differentiates those at higher risk of having an event from those at lower risk.
Model Performance:
an overall measure of accuracy that accounts for all possible outcomes, including true and false positive and true and false negative classifications. Metrics that measure this may need to be adjusted when imbalanced datasets are used.
Multicollinearity:
an analytical issue that arises when several independent variables in a model are highly correlated with each other. This can result in unstable and unreliable effect estimates of the correlated variables on the outcome of interest.
Multiple testing/comparisons problem:
a problem that arises when testing multiple hypotheses simultaneously without adjusting the alpha level of significance. As an example, if one uses an alpha level of significance (p‐value) of 0.05 and performs 20 comparisons in which none are truly significantly different, then the chance of obtaining a false positive result is: 1 – (1‐p‐value)^20^ = 1‐(0.95)^20^ = 64%. Several methods exist to correct for multiple comparisons and should be used as appropriate.
Natural language processing:
a form of artificial intelligence in which algorithms can process, analyze, and generate data encoded in human language.
Observational Health Data Sciences and Informatics (OHDSI):
an interdisciplinary international network with the mission to encourage collaboration and open‐source solutions to leverage the value of observational data. It aims to empower observational health data sciences by deploying common data models to promote federated analytics.
Observational Medical Outcomes Partnership (OMOP):
a common data model (see above) that enables the capture of information, such as admissions, providers, diagnoses, drugs, measurements, and procedures, in the same way across different institutions.
Overfitting:
occurs when an algorithm too closely models or ‘fits’ the source data on which it is trained. If this happens, it is not transferable and thus cannot make accurate predictions on novel data that were not a part of its original training set. Typically, overfitted models perform exceptionally well on training data and very poorly on new data.
Personalized medicine:
see precision medicine
Principal component analysis:
a computational method that reduces feature dimensionality by transforming the data into a new lower dimensional coordinate space by creating linear combinations of the original variables that maximally explain the variance of all the original variables.
Probabilistic linkage:
a method that links data for the same person from different datasets. This approach evaluates patterns of matching variable agreements and then generates weights (scores) that reflect the probability or likelihood that they relate to the same person, thus facilitating linkage for the same person from different datasets.
Precision medicine:
alternatively known as "personalized medicine", it is the approach of tailoring disease prevention and treatment to the individual, rather than the population, by accounting for differences in people's genes, environments, lifestyles, or other characteristics.
Real world evidence:
evidence derived from real world data that are accumulated from interactions of a person with their health system. These data include information derived from electronic health records, medical claims, prescriptions, diagnostic tests, registries, and digital health technologies. These real‐world data can be used in epidemiological, interventional, and precision medicine studies to derive evidence from settings outside strictly controlled trials.
Secondary data use:
the use of aggregated data initially collected for other purposes, such as for primary research studies or in electronic health records and administrative health records produced during routine clinical care, to perform an independent study that seeks to improve individual and population health, health systems, and health policy.
Semi‐structured data:
these data are an intermediary between structured and unstructured data. They do not conform to a rigid database structure but contain tags and markers that separate and define data elements and create a hierarchical structure. Examples include clinic notes with tags or defined properties, or secure messages sent between health care providers in electronic medical records databases.
Structured data:
these data are typically quantitative and are recorded and organized according to strict and rigid rules. These are typically tabular data with rows and columns that strictly define the data attributes. Examples include dates, names, and personal health numbers.
Unstructured data:
these data are typically qualitative and are recorded and organized without strict or rigid rules. These data cannot be processed and analyzed using conventional database tools and methods since they lack a predefined data model. Examples include free text clinic letters and diagnostic reports.
